# Tumor suppression by miR-31 in esophageal carcinoma is p21-dependent

**DOI:** 10.18632/genesandcancer.38

**Published:** 2014-11

**Authors:** Zhifeng Ning, Hua Zhu, Feifei Li, Qing Liu, Gefei Liu, Tao Tan, Bo Zhang, Shaobin Chen, Guanwu Li, Dongyang Huang, Stephen J. Meltzer, Hao Zhang

**Affiliations:** ^1^ Laboratory for Translational Oncology basic medicine college, Hubei University of Science and Technology, Xianning, Hubei province, China; ^2^ Department of Biotherapy and Gastrointestinal Medical Oncology, Affiliated Cancer Hospital of Shantou University Medical College, Shantou, Guangdong, China; ^3^ Tumor Tissue Bank, Affiliated Cancer Hospital of Shantou University Medical College, Shantou, Guangdong, China; ^4^ Department of Thoracic Surgery, Affiliated Cancer Hospital of Shantou University Medical College, Shantou, Guangdong, China; ^5^ Cancer Research Center, Shantou University Medical College, Shantou, Guangdong, China; ^6^ Department of Cell Biology, Shantou University Medical College, Shantou, Guangdong, China; ^7^ Department of Biochemistry, Shantou University Medical College, Shantou, Guangdong, China; ^8^ Department of Surgery, Davis Heart and Lung Research Institute, the Ohio State University Wexner Medical Center, Columbus, OH, USA; ^9^ Division of Gastroenterology, Department of Medicine, The Johns Hopkins University School of Medicine and Sidney Kimmel Comprehensive Cancer Center, Baltimore, MD, USA

**Keywords:** microRNA, miR-31, p21, esophageal squamous cell cancer, personalized medicine

## Abstract

microRNA regulation network is important for the cancer genetic heterogeneity. Relative to the increasing numbers of microRNA's targets identified, upstream regulatory mechanisms that control functional microRNAs are less well-documented. Here, we investigated the function of miR-31, a pleiotropically-acting microRNA, in esophageal squamous cell cancer (ESCC). We demonstrated that miR-31 only exerted tumor-suppressive effects in TE-7 ESCC cells, but not in TE-1 ESCC cells, although both of these cell lines harbor inactive p53. Interestingly, TE-1 cells highly expressed p21, while p21 levels were virtually undetectable in TE-7 cells, suggesting a p21-dependent mechanism of miR-31-mediated tumor suppression. Accordingly, knockdown of p21 in TE-1 cells reversed the tumor suppressive actions of miR-31. In patient ESCC specimens, real-time RT-PCR analysis revealed that expression of E2F2 and STK40, two known miR-31 target oncogenes, was negatively correlated with the expression of miR-31 in a p21-dependent manner, supporting the conclusion that miR-31 only downregulates its target oncogenes when p21 levels are low. Collectively, these data suggest a novel mechanism through which the tumor-suppressive effect of miR-31 is p21-dependent. In addition, we speculate that delivery of miR-31 could provide therapeutic benefit in the personalized management of a subgroup of ESCC patients with p21-deficient tumors.

## INTRODUCTION

Esophageal carcinoma ranks seventh in cancer incidence and sixth in cancer-related death worldwide, respectively, with esophageal squamous cell carcinoma (ESCC) accounting for 90% of all histological types of esophageal carcinomas diagnosed at advanced stages [1-3]. Even with improved surgical techniques, the median survival of ESCC patients after R0 resection (complete removal of the entire tumor, followed by microscopic examination of margins showing no tumor cells) is less than 2 years [4, 5]. Difficulty in treatment of ESCC is partly attributed to genetic heterogeneity of the disease, which is in part due to complex regulatory networks and is the challenge for personalized therapy [6-9]. Thus, the identification of regulatory pathways for stratification of this deadly disease is critical to developing personalized and precision therapeutics and enhancing survival.

microRNA network is of importance for genetic heterogeneity of diseases. microRNAs (miRs) are endogenous single-stranded non-coding RNAs ranging from 19 to 25 nucleotides in length which play important roles in epigenetic and post-transcriptional regulatory networks [10]. Considerable evidence has identified the involvement in cancer of one of these RNAs, miR-31, but its role is complicated because it can act as either a tumor suppressor or an oncogene: while it inhibits cell proliferation of serious ovarian carcinomas [11] and cell metastasis in breast cancer [12], and impairs migration of endometrial cancer cells [13] and growth of prostate cancer *in vivo* [14], miR-31 also promotes tumorigenesis in colorectal cancer [15] and cell migration and invasion in Kaposi's sarcoma [16]. In ESCC, miR-31 has also been reported to be both a promoter and an inhibitor of carcinogenesis [17-20]. While microarray screening showed miR-31 to be upregulated in ESCC *vs.* normal epithelia [17, 18], patients with high miR-31 expression levels had an improved prognosis [20]. Further work suggested that miR-31-mediated downregulation of DNA repair genes contributes to an improved prognosis of ESCC patients after radiotherapy [19]. These findings suggested that the complex action of miR-31 might reflect genetic heterogeneity among ESCC patients.

The multifaceted role of miR-31 suggests that its action depends on cellular and molecular context. Relative to its known downstream targets, there is less knowledge regarding how miR-31 is regulated by upstream mechanisms or interactions with other molecules. Interestingly, effects of miR-31 have been linked to the status of p53, the most frequently mutated gene in all cancers. miR-31 plays an inhibitory role only in tumor cells harboring mutant p53, suggesting miR-31 as a therapeutic target in patients with p53-deficient tumors [11]. Of note, p53 mutation is an early signature event in ESCC; moreover, changes in p53 status could account for context-dependent effects of many molecules [21], including microRNAs such as miR-31 [11]. However, it is unknown whether there is an association between p53 status and miR-31 in ESCC. Clarity regarding cellular mechanisms accounting for miR-31's function in cancer will be beneficial in designing tailored diagnostic and therapeutic strategies for ESCC and other malignancies.

In this study, we attempted to study the molecular mechanism underlying miR-31-mediated inhibition of p53-deficient ESCC. Surprisingly, we found that while the ESCC cell lines TE-7 and TE-1 harbored deficient p53, miR-31 only exhibited tumor-suppressive activity in the p21-low-expressing cell line TE-7, and not in p21-high-expressing TE-1 cells. However, after p21 was silenced by shRNA, the suppressive function of miR-31 was rescued in TE-1 cells. Moreover, we analyzed the correlation between miR-31 and its known target oncogenes, E2F2 and STK40, in 27 human ESCC tissues. As in our observations in cancer cell lines, the inhibitory effect of miR-31 on its targets was also p21-dependent. Our findings suggested a novel mechanism via which the tumor-suppressive function of miR-31 depends on p21 status, suggesting the p21-miR-31 pathway as a potential therapeutic target in a subgroup of ESCC patients.

## RESULTS

### Differential effects of miR-31 in ESCC cell lines harboring inactive p53

**Figure 1:**
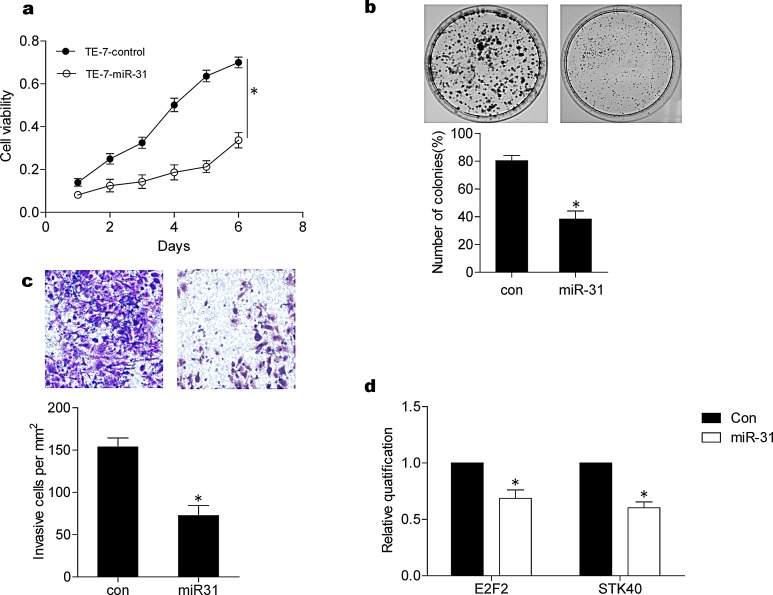
Tumor suppressive effects of miR-31 in TE-7 ESCC cells (A) Cell viability was determined by MTT assay. Transfection of miR-31 inhibited cell viability of TE-7 cells. (B) miR-31 treatment inhibited colony formation capacity of TE-7 cells by about 43% as shown in the lower panel, a representative colony formation assay was shown (upper panel). (C) Invasive ability of TE-7 cells was inhibited over 90% by miR-31 treatment. Representative pictures of invaded cells (upper panels); cell number per 1 mm2 was counted under 100 X magnification and summarized (lower panel). (D) The effects of miR-31 on E2F2 and STK40 were shown by real time RT-PCR. Transfection of miR-31 can inhibit mRNA levels of E2F2 and STK40. The data were the average of three independent experiments and presented as means ± SEM. * indicating P < 0.05 con, control.

A previous study demonstrated that miR-31 functions as a tumor suppressor only in p53-deficient cells [11]. We tested this finding in several ESCC cell lines that harbored inactive p53, including TE-1 [22] and TE-7 [23]. After overexpression of miR-31 in TE-7 cells, cell viability assessed by MTT assay decreased dramaticantly (p < 0.05) (Fig. 1A), colony formation ability tested using plate colony formation assay decreased by 43% (Fig. 1B), and cell invasion evaluated by transwell assay decreased by over 46% (Fig. 1C).

To explore molecular mechanisms underlying miR-31-mediated tumor-suppression, we examined the expression of E2F2 and STK40, two known downstream target oncogenes for miR-31 [14, 24]. Transfection and forced overexpression of miR-31 reduced E2F2 and STK40 by real-time RT-PCR (Fig. 1D), suggesting that miR-31 may suppress ESCC by downregulating target oncogenes, including E2F2 and STK40.

**Figure 2:**
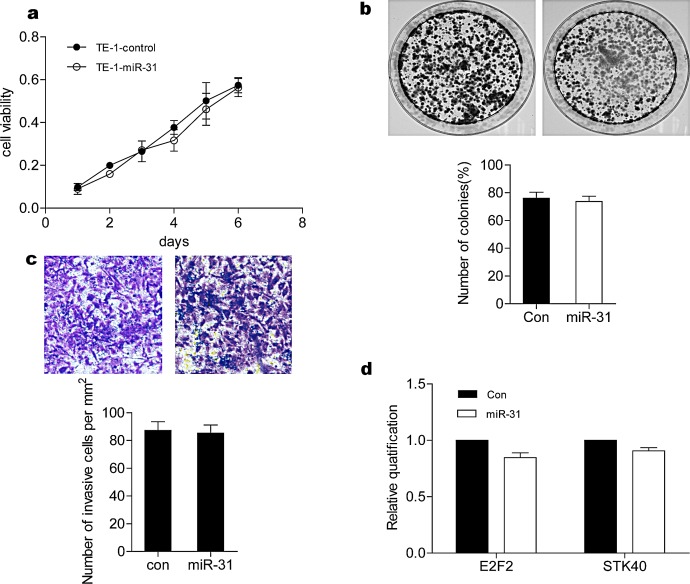
Lack of miR-31-mediated suppression in TE-1 ESCC cells (A) Cell viability was determined by MTT assay. Transfection of miR-31 did not inhibit cell growth. (B) miR-31 treatment did not inhibit colony formation capacity of TE-1 cells (lower panel). The representative figures of stained colonies (upper panel). (C) TE-1 cells invasion was not inhibited by miR-31 treatment. Representative pictures of invaded cells (upper panels), cell number per 1 mm2 was counted under 100 X magnification and summarized (lower panel). (D) Effects of miR-31 on E2F2 and STK40 were shown by real time RT-PCR. Transfection of miR-31 did not reduce E2F2 and STK40 mRNA levels. The data were the average of three independent experiments and presented as means ± SEM with * indicating P < 0.05. con, control.

To our surprise, transfection of miR-31 had no effect on TE-1 cells in terms of cell growth, colony formation, or invasion (Fig. 2A-C), nor did miR-31 downregulate E2F2 or STK40 (Fig. 2D). These results suggested that even though TE-1 cells harbor inactive p53, they express other factor(s) that inhibit miR-31 function.

### p21 inhibits the effects of miR-31

**Figure 3:**
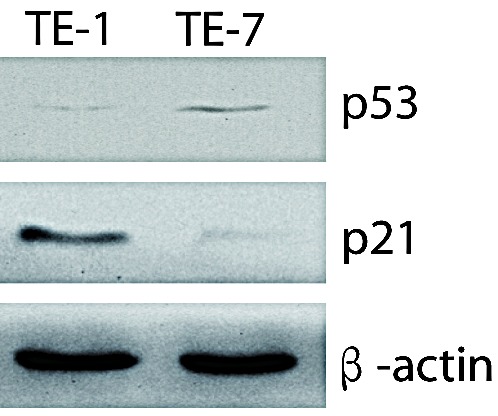
p21 was highly expressed in TE-1 cells and cannot be detected in TE-7 cells Western blot of endogenous p21 and p53 expression in TE-1 and TE-7 cells was shown. β-actin was used as internal control.

**Figure 4:**
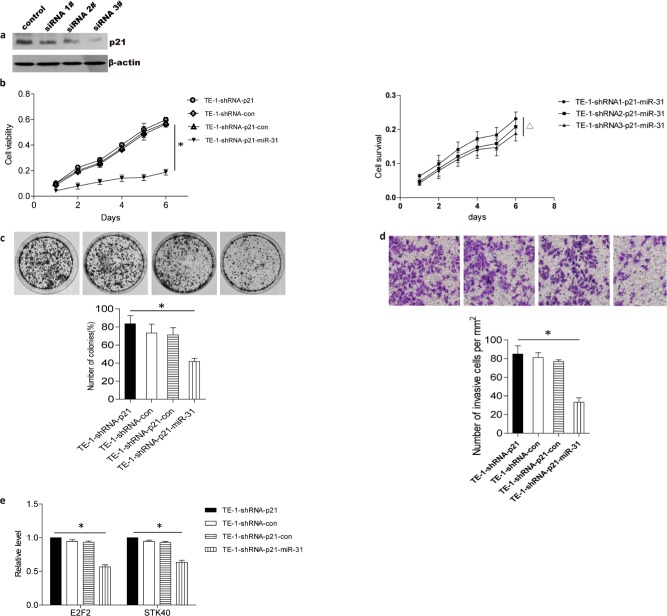
Inhibitory effects of miR-31 can be rescued in TE-1 cells by p21 shRNA treatment (A) p21 expression was silenced by shRNA in TE-1 cells, as demonstrated by western blot. 3# shRNA was selected to do the following experiment. (B) MTT assay showed silencing of p21 in TE-1 cells permitted miR-31 overexpression to inhibit proliferation (left). Moreover, suppressive effect of miR-31 depended on silenced effect of p21 in TE-1 (right). (C) Colony formation assay indicated overexpression of miR-31 inhibited colony formation of TE-1 after p21 silencing. (D) Cell invasion assays showed overexpression of miR-31 inhibited invasion of TE-1 after p21 silencing. (E) miR-31 downregulated miR-31 target genes, E2F2 and STK-40, after p21 silencing. * indicating P < 0.05. Statistical comparisons were made between TE-1-shRNA-p21-con vs. TE-1-shRNA-p21-miR-31, Δindicating P < 0.05. Statistical comparisons were made between TE-1-shRNA1-p21-miR-31 and TE-1-shRNA2-p21-miR-31 or TE-1-shRNA2-p21-miR-31 and TE-1-shRNA3-p21-miR-31. Data were average of three independent experiments and was presented as means ± SEM with * indicating P < 0.05. con, control. *, TE-1-con-shRNA-con v.s. TE-1-miR-31-shRNA-p21.

Because miR-31-mediated tumor suppression was previously shown to depend on p53 deficiency, but our miR-31-resistant TE-1 cell line was also p53-deficient, we tested TE-1 and TE-7 cells for expression of the p53 target protein p21. p21 was highly expressed in TE-1 cells, but undetectable in TE-7 cells (Fig. 3), suggesting that p21 could be responsible for inhibition of miR-31-mediated tumor suppression. To test this hypothesis, we transfected p21-specific shRNA into TE-1 cells. As shown in Fig. 4A, p21 shRNA reduced endogenous expression of p21 by 85%. Thus, if p21 inhibited tumor suppressor function of miR-31, one would expect that p21 shRNA treatment would sensitize TE-1 to miR-31. Indeed, miR-31 inhibited viability, colony formation, and invasion of TE-1 cells following shRNA-mediated knockdown of p21 (Fig. 4B-D). Moreover, the suppressive effect of miR-31 on cell survival depended on silencing of p21 in TE-1 cells (Fig. 4B). Real-time RT-PCR also showed that miR-31 was able to downregulate the expression of E2F2 and STK40 after p21 shRNA treatment (Fig. 4E), further demonstrating that the tumor-suppressive effects of miR-31 were dependent on reduced p21 expression.

### Expression of p21, miR-31, and miR-31-target genes in ESCC patients

**Figure 5:**
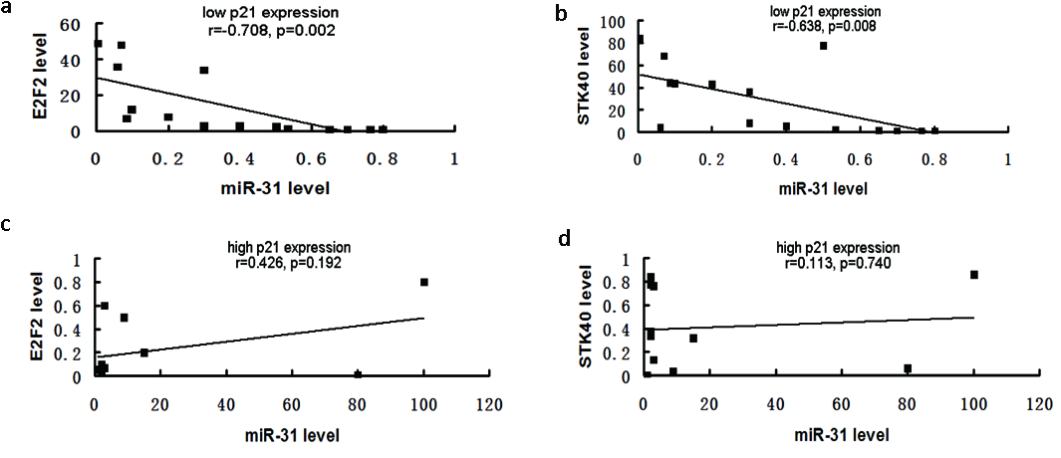
Correlation between miR-31 and its targets, E2F2 and STK40, in patients with ESCC was p21-dependent mRNA of biopsies from patients with ESCC were extracted and subjected to real time RT-PCR to assay the expression of miR-31, p21, E2F2 and STK40. Patients were grouped by the expression of p21. (A). When p21 expression was low, miR-31 was negatively correlated with its target genes, E2F2 and STK40, as shown by Pearson correlation analysis. (B). When p21 expression was high, miR-31 was not correlated with its target genes.

In order to validate our *in vitro* findings in patients with ESCC, we collected biopsies from 27 ESCC patients and assessed the correlation of p21, miR-31, and miR-31-target gene expression by real-time RT-PCR. Although expression of miR-31 and p21 fluctuated among the 27 ESCC tissues, there was no statistically significant correlation between expression of miR-31 and p21 in human ESCC biopsies (data not shown). However, miR-31 expression was negatively correlated with E2F2 and STK40 in biopsies with low p21 expression (Fig. 5A). When p21 expression was high, miR-31 expression did not correlate with E2F2 and STK40 expression (Fig. 5B). These results suggested miR-31 regulates the expression of its target genes in p21-deficent ESCC human tissues. Taken together, the observations in human tumor biopsies confirmed our hypothesis that the tumor-suppressive effect of miR-31 is p21-dependent.

## DISCUSSION

In this study, we found that miR-31 inhibits viability, colony formation and invasion in ESCC cells. This inhibitory effect appeared to be a result of downregulation of at least two downstream miR-31 target oncogenes, E2F2 and STK40. Of interest, miR-31 exerted its antitumor effects only in a cell line expressing low p21 levels, whereas the inhibitory effects of miR-31 in cells with high levels of p21 were largely silenced and could be rescued by p21 shRNA treatment. This finding was confirmed in 27 tumor tissues derived from ESCC patients by real time RT-PCR. Pearson correlation analysis for the expression profiles of miR-31, two target oncogenes, and p21 suggested that tumor cells with low p21 levels were candidates for future therapeutic delivery of miR-31.

miR-31 is a pleiotropic molecule that can act as either an oncogene or a tumor suppressor in different cancer types, although it is usually classified as a tumor suppressor [11, 25]. As in other cancer types, the function of miR-31 in esophageal cancer is controversial. While some reports identify its oncogenic potential in esophageal cancers [17, 18], Zhao et al. [20] found that high levels of miR-31 are associated with a better prognosis. Furthermore, Lynam-Lennon et al. [19] found that miR-31 was significantly reduced in esophageal tumors and downregulated in radio-resistant esophageal adenocarcinoma cells. Ectopic re-expression of miR-31 re-sensitizes radio-resistant cells to radiation, suggesting that miR-31 exhibits a tumor-suppressing function in esophageal cancer [19]. One explanation for the pleiotropism of miR-31 in different cancers was presented in an elegant study by Creighton et al. [11], wherein the authors demonstrated that the tumor-suppressive function of miR-31 in ovarian cancer was dependent on loss of p53, i.e., miR-31 only inhibited ovarian tumor cells expressing non-functional p53 [11]. In the present study, we found that in addition to regulation by p53, the inhibitory effect of miR-31 in ESCC was dependent on p21 deficiency. Our results suggest that the requirement for p53-deficiency in order for miR-31 to function as a tumor suppressor is likely due to a dependence on p21 deficiency.

p21 is a cyclin-dependent kinase (Cdk) regulator that controls cell division and cell fate [26]. This gene actively participates in the regulation of genes involved in growth arrest, senescence, and aging [27, 28]. p21 is known to function as a double-edged sword in cancers [29]. It is usually regarded as a repressor in cancers, since it is a downstream target of p53 and inhibits cell cycle progression, thereby inducing senescence. However, some studies actually imply that p21 is an oncogene [30, 31]. Several reports indicate that increased p21 expression is associated with tumor progression and a poor prognosis in prostate [32, 33], ovarian [34], cervical [35], breast [36] and esophageal squamous cell carcinomas [37], as well as in brain tumors [38]. In the present study, we showed that p21 regulates the tumor-suppressive function of miR-31 in ESCC. When p21 is highly expressed, miR-31 does not inhibit expression of miR-31 downstream target genes, nor does miR-31 inhibit proliferation, colony formation, or invasion. One explanation of this phenomenon is the possibility that p53 mutation led to low p21 expression and changed susceptibility to cancer treatment [39, 40]. Our Pearson correlation analysis of the expression levels of miR-31, its target oncogenes, and p21 in 27 ESCC tissues suggested that miR-31 can regulate its target genes only in p21-low patients.

Although p21 is usually regarded as a downstream effector gene of p53 and is believed to be rarely mutated [41], high-throughput gene chip assays have recently suggested that p21 expression varies among cancer cell lines and cancer tissues, including esophageal squamous cell cancers [42-44]. Moreover, p21 is regulated not only by p53, but also by other regulators such as MTA1 [45], E2F1 [46] or several nuclear receptors [47] including retinoid receptors, vitamin D receptors and androgen receptors. Thus, future studies are required for exploring the molecular mechanisms by which p21 regulates the tumor suppressive function for miR-31 and potential interventions to disrupt the p21-mediated suppression of miR-31 for treatment of ESCC patients.

**Figure 6:**
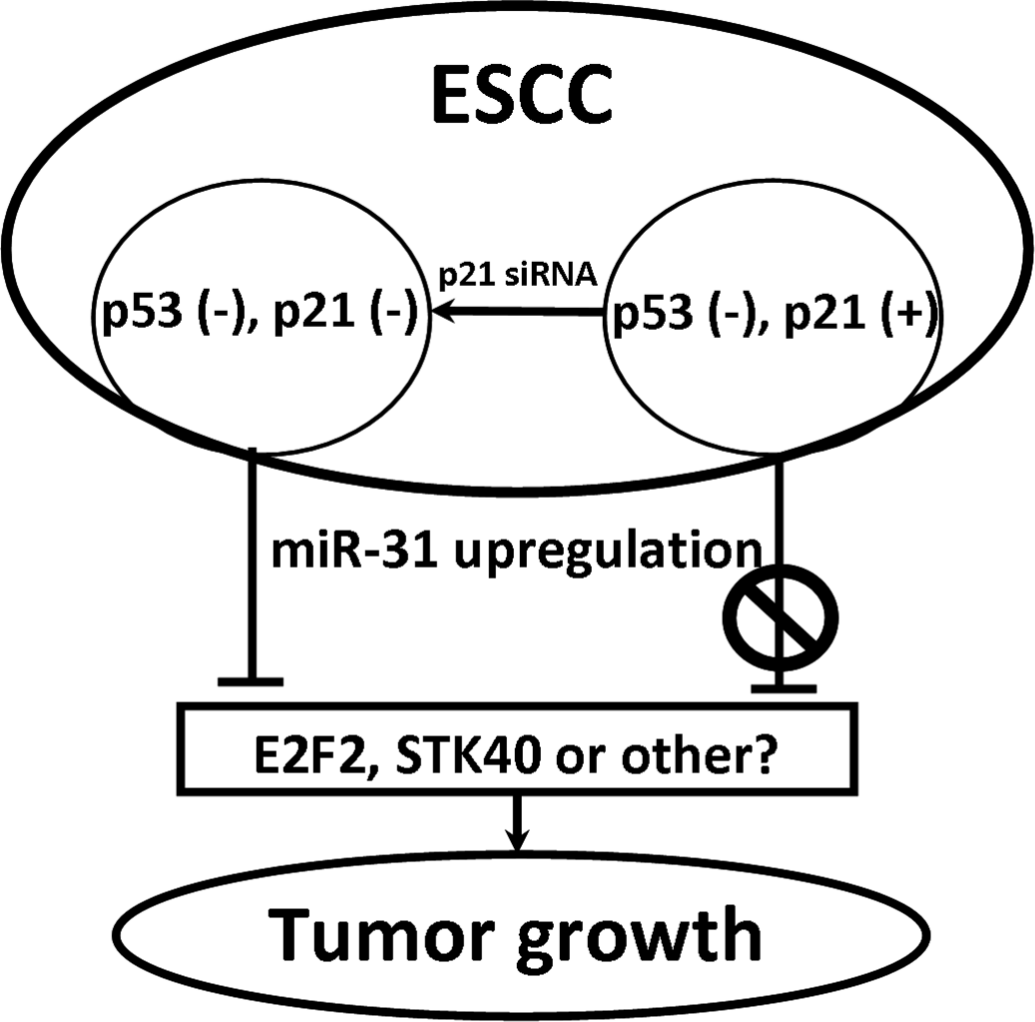
Schematic diagram of p21 dependence of tumor suppression by miR-31 in ESCC

In summary, we demonstrate that miR-31 exhibits inhibitory effects in ESCC, in a p21-dependent manner (summarized in Fig. 6), providing a novel mechanism for the tumor suppressor function of miR-31. From a translational perspective, our data suggest that in the future, miR-31 delivery therapy may benefit ESCC patients with low p21 expression, constituting a potential target for personalized medicine.

## MATERIALS AND METHODS

### Clinical specimens

Twenty-seven pairs of primary ESCC tumors were obtained from 27 patients (median age at diagnosis, 60.6 years; range from 38 to 79 years). Matched normal adjacent tissues were defined as tissues located at least 1.0 cm apart from the visible tumor lesions. Specimens were deposited in RNALater (Qiagen, Germany) or snap-frozen in liquid nitrogen and subsequently stored at −80°C. All patients underwent esophagectomy without preoperative chemotherapy or radiotherapy in the Affiliated Cancer Hospital of Shantou University Medical College between November of 2010 and May of 2011. This study was approved by the Ethics Committee of Shantou University Medical College, and written informed consent was obtained from all patients.

### Cell lines

Human esophageal squamous cell carcinoma TE-1 and TE-7 cell lines were kindly provided by Dr. X. C. Xu (UT M.D. Anderson Cancer Center, USA) and cultured in high glucose DMEM supplemented with 10% fetal bovine serum (Gibco), 100 U/ml penicillin and 100 μg/ml streptomycin at 37°C in a humidified atmosphere containing 5% CO_2_, and passaged when cells reached nearly 80% confluence.

### Overexpression of miR-31

TE-7 or TE-1 cells were transfected in six-well plates (2.5×10^5^ per well) using 7.5 μL Lipofectamine 2000 transfection reagent (Invitrogen) and 3 μg hsa-miR-31 mimic (90 nmol/L; Dharmacon) according to manufacturer's instructions. Control groups of cells were treated with transfection reagent alone (mock transfection). Cells were harvested 48 h after transfection, and E2F2 and STK40 were tested by real time RT-PCR. For functional assays including proliferation, colony formation and invasion measurements, CMV-TurboRFP-miR-31-IRES-puro (Open Biosystems) was packaged and used to infect the TE-7 or TE-1 cell lines, according to the manufacturer's instructions. The DNA plasmid carrying a non-targeting sequence (Open Biosystems) was used as a negative control. Forty-eight hours post-transfection, virus-containing media was filtered (0.45 μm) and added onto TE-7 or TE-1 cells with appropriate dilution in the presence of polybrene (8 μg/mL). The virus-containing media was changed with fresh cell medium 6 h post-infection. The infection efficiency was measured by examining the cells under fluorescence microscope and was determined to be >95%.

### shRNA knockdown of p21 in TE-1 cells

Stable transfection was performed at about 80% confluence in 24-well plates using Lipofectamine LTX and Plus Reagents (Invitrogen) according to the manufacturer's instructions. Briefly, a total of 2×10^5^ TE-1 cells were inoculated into each well in high glucose DMEM containing 10% FBS without antibiotics. p21 shRNA (shp21) or control shRNA (0.1 mg) (mock) (Santa Cruz Biotechnology) vectors were transfected with 0.5 ml of Plus Reagents and 1.25 ml of Lipofectamine LTX. Interference sequences for p21 shRNA were listed at supplementary table 1. After transfection, cells were isolated in culture medium containing 2 mg/ml puromycin (Invitrogen). After 3 to 4 weeks, resistant cell colonies were isolated and transferred to 6-well plates, and gradually expanded to 10-cm dishes. At 90% confluence, western blot analyses were performed to assess the efficiency of p21 knockdown.

### MTT assay

Cell proliferation was measured with a colorimetric assay reagent, thiazolyl blue tetrazolium Bromide (MTT, Sigma-Aldrich, China). Absorbance was read at 590 nm with a reference filter of 620 nm.

### Transwell invasion assay

Cell invasion assays were performed using transwells. Briefly, 1×10^4^ cells were inoculated in a 24-well transwell unit on polycarbonate filter with 8 μm pores (Costar, Cambridge, MA) coated with Matrigel (Becton Dickinson, Franklin Lakes, NJ). After 24-h incubation at 37oC, cells that had passed through the filter were stained with Giemsa and scored for the number.

### Colony formation assay

For colony formation, cells (2×10^3^) were trypsinized and cultured in 60-mm culture dishes. The dishes were incubated for two weeks, then colonies were stained with 0.1% crystal violet, then photographed and counted.

### Western blot assay

As previously described [48], cells were lysed in RIPA buffer. The proteins were separated by SDS-PAGE and then transferred to PVDF membranes (Millipore, Bedford, MA). Blots were probed with antibodies against p53 (1:500, Santa Cruz, Dallas, TX), p21 (1:1000, Cell Signaling, Danvers, MA), β-actin (1:5000, Sigma, St. Louis, MO). After washing, blots were incubated with horseradish peroxidase-conjugated secondary antibodies and visualized using an enhanced chemiluminescence kit (Pierce, Rockford, IL).

### Real time RT-PCR assay

Cells or tissues were harvested with Trizol Reagent (Invitrogen, Carlsbad, CA, USA) and total RNA was isolated according to the manufacturer's instructions. cDNA synthesis was performed using the Superscript III RT-PCR kit (Invitrogen). Real-time PCR was performed using a Cepheid SmartCycler II (Sunnyvale, CA, USA) with gene-specific real-time PCR primers. Specifically, stem-loop real-time RT-PCR was used to analyze the expression of miR-31. Relative quantification (RQ) of selected genes and miR-31 expression was normalized with respect to GAPDH and U6 respectively. Corresponding adjacent esophageal tissues were used as calibrator samples. The expression of the target gene was calculated using the equation 2-ΔCt, where ΔCt = (Ct target gene – Ct reference gene). The relative expression of target genes in carcinoma tissue was calculated by 2-Δ ΔCt, where Δ ΔCt = (ΔCt target gene in the tumor tissue – ΔCt target gene in the adjacent normal tissue). Data were presented as log10 of the relative quantification equal to the fold-change of gene expression in ESCC tissue compared to its corresponding adjacent esophageal tissue. High or low p21 expression was defined as its expression was higher or lower compared to corresponding adjacent esophageal tissue. The primers for PCR are list in supplementary table 2.

### Statistical analysis

Comparisons of real time data were analyzed by the unpaired t test, whereas qualitative data were analyzed by the chi-square test. Correlation was determined by Pearson correlation analysis. All statistical analyses were performed and visualized by GraphPad Prism 5.0. A P < 0.05 was considered statistically significant. All experiments were performed in triplicate and repeated twice.

## SUPPLEMENTARY TABLES



## Abbreviations

ESCC: esophageal squamous cell cancer
miR-31: microRNA-31
p21: cyclin-dependent kinase inhibitor 1
E2F2: E2F transcription factor 2
STK40: serine/threonine kinase 40
RT-PCR: reverse transcriptase-polymerase chain reaction
