# Supplementary material for: Tumor suppression by miR-31 in esophageal carcinoma is p21-dependent
Source: Genes Cancer. 2014 Nov;5(11-12):436–44. doi: 10.18632/genesandcancer.38 (PMC4279440; doi:10.18632/genesandcancer.38)
Supplement: Supplementary file 1 [file ganc-05-436-s001.pdf]

Tumor suppression by miR-31 in esophageal carcinoma is p21-dependent – Ning et al

Supplementary table 1. Interference sequence of p21 shRNA

shRNA	Sequence (3'-5')
shRNA 1	GAGGGUGUUACGACUUAUAGAGAACTAUAUUCAGCAUUGUGGGAG
shRNA 2	GGAGGGUGUUACGACUUAUGAGAACTUAUUCAGCAUUGUGGGAGG
shRNA 3	CCCUCAUCAACAGAAAGGAGAGAACTAGGAAAGACAACUACUCCC
Negative control	GCAAGCTGACCCTGAAGTTGAGAACTTTGAAGTCCCAGTCGAACG

Supplementary table 2. List of primers used in real-time RT-PCR analysis

Name of the gene	Accession No	Forward Seq. (5'-3')	Reverse Seq. (5'-3')	Amplicon size (bp)
GAPDH	NM_002046.4	GCACCGTCAAGGCTGAGAAC	TGGTGAAGACGCCAGTGGA	138
E2F2	NM_004091.3	TGAGCTTCAAGCACCTGACTGA	TTGCCAACAGCACGGATATC	82
STK40	NM_032017.1	CGTGCACAGAGACCTGAAGCT	GAGGCAGAAGTTGGTGATGGTT	79
U6	NM_004394.1	CTCGCTTCGGCAGCACA	AACGCTTCACGAATTTGCGT	106
miR-31	NR_029505.1	GCCGCAGGCAAGATGCTGGC	CAGTGCAGGGTCCGAGGT	71
